# Sample Entropy Analysis of EEG Signals via Artificial Neural Networks to Model Patients' Consciousness Level Based on Anesthesiologists Experience

**DOI:** 10.1155/2015/343478

**Published:** 2015-02-08

**Authors:** George J. A. Jiang, Shou-Zen Fan, Maysam F. Abbod, Hui-Hsun Huang, Jheng-Yan Lan, Feng-Fang Tsai, Hung-Chi Chang, Yea-Wen Yang, Fu-Lan Chuang, Yi-Fang Chiu, Kuo-Kuang Jen, Jeng-Fu Wu, Jiann-Shing Shieh

**Affiliations:** ^1^Department of Mechanical Engineering and Innovation Center for Big Data and Digital Convergence, Yuan Ze University, Chung-Li, Taoyuan 32003, Taiwan; ^2^Department of Anesthesiology, College of Medicine, National Taiwan University, Taipei 100, Taiwan; ^3^Department of Electronic and Computer Engineering, College of Engineering, Design and Physical Sciences, Brunel University London, Uxbridge UB8 3PH, UK; ^4^Department of Anesthesiology, National Taiwan University Hospital, Yuan Lin Branch, Yuan Lin 64041, Taiwan; ^5^Department of Anesthesiology, Shuang Ho Hospital, Taipei Medical University, Taipei 23561, Taiwan; ^6^Missile & Rocket Systems Research Division, National Chung-Shan Institute of Science and Technology, Longtan, Taoyuan 32500, Taiwan; ^7^Center for Dynamical Biomarkers and Translational Medicine, National Central University, Chung-Li 32001, Taiwan

## Abstract

Electroencephalogram (EEG) signals, as it can express the human brain's activities and reflect awareness, have been widely used in many research and medical equipment to build a noninvasive monitoring index to the depth of anesthesia (DOA). Bispectral (BIS) index monitor is one of the famous and important indicators for anesthesiologists primarily using EEG signals when assessing the DOA. In this study, an attempt is made to build a new indicator using EEG signals to provide a more valuable reference to the DOA for clinical researchers. The EEG signals are collected from patients under anesthetic surgery which are filtered using multivariate empirical mode decomposition (MEMD) method and analyzed using sample entropy (SampEn) analysis. The calculated signals from SampEn are utilized to train an artificial neural network (ANN) model through using expert assessment of consciousness level (EACL) which is assessed by experienced anesthesiologists as the target to train, validate, and test the ANN. The results that are achieved using the proposed system are compared to BIS index. The proposed system results show that it is not only having similar characteristic to BIS index but also more close to experienced anesthesiologists which illustrates the consciousness level and reflects the DOA successfully.

## 1. Introduction

Accurate and noninvasive monitoring of depth of anesthesia (DOA) is taken more and more seriously since it becomes one of the anesthetic techniques that are frequently used in the surgery operation [1]. However, anesthesiologists have multiple inconsistent definitions of the anesthetic state and have no standard measurement to assess it. Although many devices and techniques are developed for detecting the DOA directly using human physiological signals like heart rate (HR), blood pressure (BP), and electroencephalogram (EEG) [2–6], the patient can be controlled by manipulating the monitored values, but the response is often delayed. Also, some direct measurements cannot provide sufficient information of the autonomic nervous system (ANS) and central nervous system (CNS), which are related to the DOA [7]. For the reason of avoiding intraoperative awareness, such physiological signals are considered one major topic when accessing the DOA, with the main reaction of anesthetic agent happened in the brain. Therefore, in the search for such a reliable indicator of DOA among these physiological signals, EEG signals having the ability to express brains activities and reflecting the human awareness have become one of indispensable and more intuitively roles when investigating the DOA [7, 8].

As we know, our vital signs, especially for EEG signal which is quite small in the microvolt level, in operating theatre, are easy contaminated by noise, for example, diathermy effect which is caused by electrosurgical knife between 300 kHz and 3 MHz. Also, the movement of the patient during surgical operation easily induces the artifacts to interfere the vital signs. Therefore, to get rid of noise and artifacts and to decompose this vital sign into more physiological meaning are fundamental part of this presignal processing. Fortunately, EMD has been proposed in 1998 as an innovative method applied to decompose intrinsic mode functions (IMFs) from a complex time series [9]. Recently, ensemble EMD (EEMD) has been proposed for dealing with mode-mixing problems [10]. Moreover, multivariate EMD (MEMD) has been proposed for dealing with multivariate parameters and solving the mode-mixing for adding noise as well. Also, MEMD can reduce the iteration times for getting rid of noise adding into the original signals [11, 12]. Therefore, the features of MEMD can be possibly applied to noise and artifacts reduction.

The bispectral index (BIS) monitor, which introduced by Aspect Medical Systems, Inc., in 1994, is the most widely used system to assess the DOA [13–15]. BIS is mainly derived from the EEG signals; specifically the frontal electrodes provide a measure of the patient's level of consciousness by calculating dimensionless number. The calculated BIS index reflects the awake state and provides the activity of brain, ranged from 0 to 100 (40~60: adequate general anesthesia; under 40: deep hypnotic state) [16]. In many previous studies, BIS has been proved as one reliable indicator when assessing the DOA, as described the level of consciousness of brain during anesthesia. However, a study found that patients can become aware even when BIS values are within the target range (i.e., 40 to 60) and thus concluded that the BIS monitoring should not be used as part of standard practice of anesthesia [17].

In addition to BIS, entropy, another way is tested by researchers to access the DOA through EEG signals. A commercial product designed by Datex-Ohmeda division of General Electrics is called entropy module. Two entropy indices, that is, response entropy (RE) and state entropy (SE), are calculated and displayed simultaneously [18]. However, RE and SE are based on spectral entropy which might miss important information due to the use of FFT in the algorithm [19].

Attention, therefore, has turned to entropy analysis in time domain. The concept of entropy when applied to bioinformatics is that an entropy value can address the system randomness and predictability through different calculation algorithms. The entropy value decreases when the patient is at anesthetic status because the EEG signals have a lower complexity, and vice versa [20]. Entropy analysis algorithms are used in conjunction with DOA using approximate entropy (ApEn) [21–24] and sample entropy (SampEn) [25, 26]. In previous study [27, 28], SampEn has been proved better than ApEn. Moreover, these two algorithms are proposed to monitor the DOA of patients during surgeries, which show that the SampEn is more adaptive to the real time detection and has better ability in accessing the level of consciousness of patients during surgery [29–31]. The only drawback of SampEn is the calculated entropy value which is ranged from 0 to 3 variously. Furthermore, it is without any medical support to rely on and to define the value of consciousness is more suitable when scoring the DOA in a surgery. Therefore, the purpose of this paper is to estimate and define the DOA through SampEn analysis via artificial neural network (ANN).

ANN, inspired by animal's central nervous systems, is a model of computation based on the structure of biological neural networks [32]. It is one of many artificial intelligent methods that can actually learn from observing data sets and provide most accurate result, through matching the input and output data [33]. The application of ANN had been used in many fields such as science, industry, commercial product, and information systems [34, 35]. In this research, a system is developed for assessing the DOA through combining the SampEn via ANN. Hence, experienced anesthesiologists are asked to plot the score of “the state of anesthetic depth” along the time, based on clinical recordings and their own clinical experiences. This score is called the expert assessment of conscious level (EACL). Then, the SampEn is trained, validated, and tested using EACL via ANN model. The testing results of the ANN model output are compared with commercial product of BIS values.

## 2. Materials and Methods

### 2.1. Data Source

The original signals data are collected from 64 patients (i.e., 23 males and 41 females), aged 22–79 years under surgery with general anesthesia at National Taiwan University Hospital (NTUH). The duration of anesthesia ranges from half to three hours. The types of general anesthesia can be divided into three groups which are (1) general anesthesia with tracheal intubation using sevoflurane or desflurane of 32 patients; (2) general anesthesia with laryngeal mask airway (LMA) using sevoflurane or desflurane of 23 patients; and (3) total intravenous anesthesia with propofol of 9 patients. For the data recording, EEG, ECG, BP, and so forth signals are recorded by physiological monitor (Philips Intellivue MP60) and saved in a portable computer. In this research, the EEG signals that are recorded by BIS sensor are also used to measure the BIS index. The Institutional Review Board of NTUH had approved the present study, and personal informed consents are obtained from the participants before the operation.

### 2.2. Multivariate Empirical Mode Decomposition

In the operating theatre, EEG signals are usually slight and easy to be interfered by other signals like electromyography (EMG), electrooculogram (EOG), and electrosurgical unit (ESUs) [36]. In this research, previous research is referred, which using the multivariate empirical mode decomposition (MEMD), to filter the original EEG signals before doing the SampEn analysis (i.e., IMF 2 and IMF 3 are considered) [26].

MEMD, which improved from empirical mode decomposition (EMD), is proposed by Rehman and Mandic in 2010 [11]. EMD algorithm, proposed by Huang et al. in 1998 [9], can decompose the original signal into different intrinsic mode functions (IMFs), expressed as follows:

(1) $$\begin{array}{c} X\left(t\right)=\overset{n}{\underset{i=1}{\sum }}C_{i}\left(t\right)+r_{n}\left(t\right), \end{array}$$

where *X*(*t*) is the original signal in time domain, *C*_*i*_(*t*) is the IMF, and *r*_*n*_(*t*) is the residue. Through choosing the different IMFs and combine them into different combination, the noise can be reduced when the signal is reconstructed. However, mode-mixing problem is an existing problem in EMD that causes some fast intermittent signals riding on a slow oscillating wave [37]. MEMD, which solved the problem, and the noise-assisted MEMD were further proposed in 2011 [11, 12]. N-A MEMD not only can deal with multichannel signals but also can solve the mode-mixing problem by adding white Gaussian noise to the channels. The computation of N-A MEMD is listed as follows:

(2) $$\begin{array}{c} m\left(t\right)=\frac{1}{K}\overset{K}{\underset{k=1}{\sum }}e^{\theta k}\left(t\right), \end{array}$$

where *e* ^*θk*^(*t*) is the multivariate envelope curves of the whole set of direction vector, *K* is the length of the vectors, and *m*(*t*) is calculated by means of the multivariate envelope curves.

### 2.3. Sample Entropy

Entropy, as a concept that a value would be reasonable characterized from a series in an ordered system, can be described as kind of index of regularity or the degree of randomness. The entropy will have a higher value if the number of sequences in a series is more complicated or without ordered, and vice versa.

Sample entropy (SampEn), developed by Richman and Moorman [27], improved from approximate entropy (ApEn) and reduced the bias that caused by self-matching. The function of SampEn, listed below, is the negative of logarithmic that two similar sequenced of *m* consecutive data points remain similar at the next point (*m* + 1) or not

(3) $$\begin{array}{c} S_{E}\left(m,r,N\right)=-\ln\left(\frac{C_{m+1}\left(r\right)}{C_{m}\left(r\right)}\right), \end{array}$$

where *C*_*m*_ is defined as follows:

(4) $$\begin{array}{c} C_{m}\left(r\right)=\frac{\left\{\text{number}\;\text{of}\;\text{all}\;\text{probable}\;\text{pairs}\;\left(i,j\right)\text{with}\;\left|x_{i}^{m}-x_{j}^{m}\right|<r,i\neq j\right\}}{\left\{\text{number}\;\text{of}\;\text{all}\;\text{probable}\;\text{pairs},\;\text{i}.\text{e}.\;\left(N-m+1\right)\left(N-m\right)\right\}}. \end{array}$$

Therein, |*x*_*i*_^*m*^ − *x*_*j*_^*m*^| denotes the distance between points *x*_*i*_^*m*^ and *x*_*j*_^*m*^ in the space of dimension, *m*, *r* represent the tolerable standard deviation of the time series, and *N* is the length of the time series. Many theoretical researches have proved that SampEn has a better statistical validity for *m* = 1 or 2 and the range of *r* around 0.1 to 0.25. Therefore, we set the parameter *m* = 2 and *r* = 0.1 in this research accordingly [26].

### 2.4. Artificial Neural Network

ANN, a humanlike system of nerve structure of brain, is an intelligent method can similarly imitate how the brain works by using parallel computing model. Through using different learning rules, trained by related-sample data set, and having error correction, the corresponding model of ANN can be constructed. There are three learning rules in ANN generally: supervised learning, reinforced learning, and unsupervised learning [38]. In this research, a backpropagation neural network (BPNN) which is one of popular ways in supervised learning rule was used. The model of ANN system can diagnose, estimate, and provide the prediction of ideal consequences through the prelearning experience when facing a new related problem. The effort had led the ANN to be used in many fields of study, such as engineering, ecology, biology, and agriculture.

### 2.5. Expert Assessment of Consciousness Level

The whole course of anesthesia is observed and recorded by two research nurses. Any clinical events and signs which were possibly related to the depth of anesthesia are carefully recorded. The recorded information includes (1) heart rate and arterial blood pressure measurements along the whole course, (2) the anesthetic events, including induction of anesthesia, tracheal intubation and extubation, adding and reversal of muscle relaxant drugs, and managing and suctioning of the airway, (3) the surgical events, including the start and end of surgical procedure, and the occurring of any specific noxious stimulus, (4) the clinical signs of the patients, including any kinds of movement and unusual responses, and the arousability during the induction and emergence period of anesthesia, and (5) any other events that were considered to be relevant.

Five experienced anesthesiologists are asked to plot the score of “the state of anesthetic depth” along the time, based on these recordings and their own clinical experiences. This score is called the expert assessment of conscious level (EACL). These anesthesiologists made the decision solely by the recordings mentioned above in their hands and did not contact with the real patients. This is a simulation of the real clinical situation. The EACL score is ranged as 0 to 100, set parallel to the BIS index. Value of 100 represents totally awake state, and 0 represents the contrary. Values of 40 to 60 are defined as “the anesthetic depth suitable for surgery,” like the BIS index. Assigning an EACL value of below 40 means that the anesthesiologist felt the anesthesia is too deep and he/she tended to decrease the dose of anesthetic agent if he/she is in the real scene. On the contrary, assigning an EACL value of above 60 means that the anesthesiologist decided the anesthesia is insufficient for the surgical stimulation. The original EACL curve is handmade and plotted on recording papers; these are scanned and digitized into numerical data. Through using the EACL curve as standard, the results from SampEn with medical standard are used to train the ANN model. To achieve the purpose, a combination of SampEn result (ranged from 0 to 3) with medical corroboration can provide a more valuable reference to the DOA for clinical researchers. As we know, the experienced anesthesiologists (i.e., attending physicians) have been trained quite a long time. This pattern of their plot could be acted as a gold pattern for determining DOA. Then, the ANN can be applied to train, validate, and test for constructing an ANN model. If the more data are accumulated, the retraining ANN model can be more accurate.

## 3. Results

**Figure 1:**
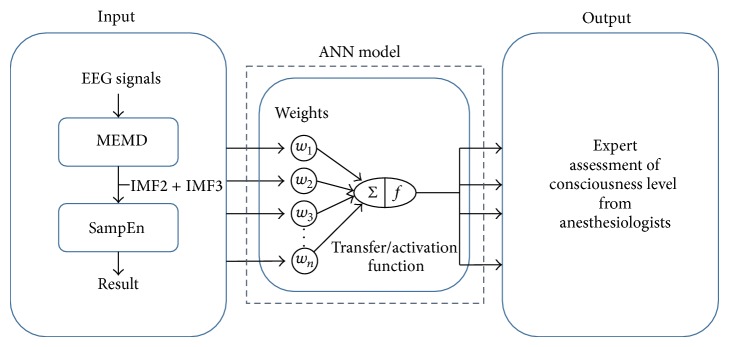
Flowchart of ANN model.

In this research, the original EEG signal is firstly filtered using the N-A MEMD, ideal combination of IMF 2 + IMF 3 is considered according to a previous study [26]. Secondly, in order to be consistent with BIS monitor that output an index every 5 seconds, here, every 625 EEG data point is used as one window size to calculate the SampEn (the sampling frequency is 125 Hz for EEG recording). Lastly, after the results of SampEn of whole operation are calculated, through the known results of SampEn and the known values of EACL, the ANN model can be constructed. A flowchart of ANN construction is shown in Figure 1.

**Figure 2:**
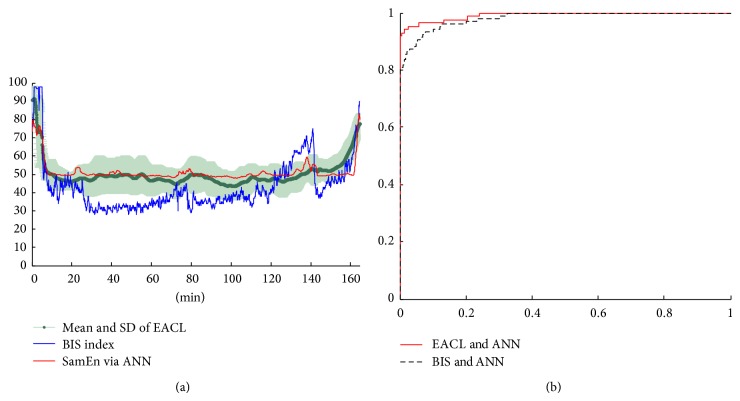
A schematic diagram of one of the testing surgery events (a) EACL, BIS index, and SampEn via ANN, (b) ROC curve.

For the ANN model construction, the mean value of EACL from five experienced anesthesiologists is used as the gold standard to train and build the transfer/activation function with different weight inside the ANN model for SampEn. The SampEn results from EEG signals are used as the input and the mean value of EACL is used as the output of ANN. Among all of 64 cases that are collected so far, 30 cases are used as training data, and 10 cases are used as validation data for setting up the ANN model (Figure 1). The rest of the 24 cases of the testing data to test this ANN model can be considered as new unknown operation events using the input values of SampEn. Through the preconstructed ANN model, the anesthesiologists assess the EACL and compare to the ANN predicted DOA using the SampEn. A schematic diagram of one of the testing surgery events is shown in Figure 2(a), where the red curve (SampEn via ANN) is presenting the prediction of EACL.

In order to compare the performance results from SampEn via ANN with BIS and EACL when assessing the consciousness level, correlation coefficient and the mean square error (MSE) are calculated. The results of correlation coefficient and MSE from the 24 testing data are listed in Table 1. The mean value and standard deviation of correlation coefficient of testing data is 0.73 ± 0.17 (EACL versus SampEn via ANN), 0.62 ± 0.19 (EACL versus BIS), and 0.71 ± 0.18 (BIS versus SampEn via ANN). The mean value and standard deviation of MSE is 10.19 ± 4.13 (EACL versus SampEn via ANN), 13.93 ± 4.38 (EACL versus BIS), and 13.24 ± 3.99 (BIS versus SampEn via ANN).

Besides, the receiver operating characteristic curve (ROC) is also calculated, using the EACL and BIS curve as standard to investigate the effectiveness of discriminate rate of SampEn via ANN in this research. ROC is often used in medical field when diagnosing diseases. By calculating the area under the ROC curve (AUC), the probability to distinguishing between awake and anesthesia state can be realized. The threshold to separate the condition of anesthesia and awake is set as 65. One of the testing surgery events of the ROC curve is shown in Figure 2(b). The results of the area under ROC curve (AUC) of testing 24 patients are listed in Table 2. The mean value and standard deviation of AUC is 0.953 ± 0.07 (EACL versus SampEn via ANN) and 0.949 ± 0.08 (BIS versus SampEn via ANN).

## 4. Discussion

The EEG represents electrical activity of the cerebral cortex derived from summated inhibitory and excitatory postsynaptic activity. The BIS is derived from EEG signals. It is calculated from a multivariate logistic regression analysis from a collected database of EEG recordings of large population size [13, 39]. However, the reliability of BIS has been questioned [17], in part because its calculation does not rely on any underlying physiological model of how the brain functions nor how awareness is generated. In addition, during ketamine, dexmedetomidine, N_2_O, and xenon anesthesia, the BIS does not perform well [40]. Also, another famous commercial product (i.e., AAI index of auditory evoked potentials (AEP) monitor) for monitoring DOA has turned to evoked potentials in brain signals which more directly reflects the subjective clinical signs that anesthesiologists have used over the years to assess their patients during anesthesia. However, a clinical comparison of three different anesthetic depth monitors (i.e., bispectral index (Aspect Medical), AAI auditory evoke potential (Danmeter), and entropy (Datex-Ohmeda)) during cardiopulmonary bypass of 21 patients has found more than a third of the paired indices agreed poorly or were even contradictory [41]. The main reason of causing this problem is these three commercial products are all measuring EEG signals of cerebral cortex. However, some drugs for anesthetics may act on thalamus and brain stem [42]. When the drugs are acting on these two sides, the EEG monitors become not so useful. Hence, another vital sign, which can represent these two sides change, can be considered into determining DOA. In our previous study [43], a short-term parameter of heart rate variability (HRV) is used to distinguish awake from isoflurane anesthetic states because ECG is controlled by brainstem of sympathetic and parasympathetic nerves. Hence, the HRV, if suitably processed, can play roles in monitoring of anesthetic depth. Moreover, due to the high performance of parallel computing and mature embedded system technology playing the key role of biomedical engineering applications, it allows the implement more complicated signal processing algorithms into general anesthesia and to dig out deep knowledge hidden behind these signals in terms of interpretation of more accurate DOA. Therefore, in the search for reliable monitor of general anesthesia, it needs to consider multiparameters. However, in considering noninvasive signals and real time analysis for beneficial patients and anesthesiologists, the EMG, ECG, BP, EEG, and SpO2 would be a good candidate for representing the DOA.

Although the constructed ANN model so far seems to have great efficiency for the consciousness level detection and further reflect the DOA, there still exists space for improvements. During the construction of the ANN model, the training part, probably better ANN model can be realized by increasing the training events and making the model meets every patient's condition more precisely. Also, another method for improving the generalization of an ANN model is ensemble ANNs method which is to avoid overfitting and to make sure the generalization of an ANN model [44, 45]. Besides, the mean values of EACL (gold standard) used to supervise the input SampEn in the ANN model are validated by different anesthesiologists in order to minimize personal error. Although in the experiment, followed the previous study of the MEMD filter part with the combinations of IMF 2 + IMF 3, different combinations can be tested in the future. Nevertheless, multiscale entropy (MSE) and its extended version multivariate multiscale entropy (MMSE) [46–48] which ameliorated from SampEn analysis and have outstanding performance are also worth to investigate in subsequent research. Finally, these analyses will be tested and tried to retrain the constructed ANN model, by increasing the experimental data and accumulating more precious EACL. With more testing data, better validation can be achieved in comparison to EACL and the BIS index. This is hoped to help clinical researcher step further to measure the DOA and avoid critical medical situations.

## 5. Conclusions

In the present study, the results of SampEn via ANN are comparable to the values EACL that are estimated by experienced anesthesiologist as well as the BIS index that is calculated by the BIS module (MP60). The correlation coefficients for both cases (EACL versus SampEn via ANN and BIS versus SampEn via ANN) are generally high and the MSEs for both are lower. Another aspect is that, from the area under receiver operating characteristic curve (AUC) result, SampEn via ANN has also shown excellent performance when detecting the state between awake and anesthesia no matter which method is used (EACL or BIS) as standard. Through the testing data, the constructed ANN model proved to be useful, and it can recognize and further transfer the input SampEn values into the prediction of EACL (SampEn via ANN) successfully.

## Figures and Tables

**Table 1:** The result of correlation coefficient and mean square error.

Event	Correlation coefficient			Mean square error		
	EACL and SampEn via ANN	EACL and BIS	BIS and SampEn via ANN	EACL and SampEn via ANN	EACL and BIS	BIS and SampEn via ANN
Patient 1	0.85	0.87	0.94	7.99	11.31	15.71
Patient 2	0.59	0.73	0.84	12.60	7.20	13.54
Patient 3	0.55	0.67	0.80	11.94	11.58	11.31
Patient 4	0.75	0.60	0.54	7.07	18.17	11.62
Patient 5	0.73	0.82	0.69	9.88	7.49	12.31
Patient 6	0.75	0.39	0.51	11.76	14.72	17.08
Patient 7	0.85	0.83	0.80	6.74	15.77	14.15
Patient 8	0.78	0.88	0.87	7.26	13.99	14.09
Patient 9	0.60	0.40	0.50	10.09	17.39	18.61
Patient 10	0.87	0.65	0.55	9.93	12.58	12.84
Patient 11	0.67	0.51	0.82	19.43	14.44	20.03
Patient 12	0.79	0.68	0.82	12.55	10.56	10.77
Patient 13	0.85	0.75	0.78	4.09	11.95	13.03
Patient 14	0.89	0.80	0.90	9.61	15.32	7.38
Patient 15	0.72	0.75	0.76	10.36	9.63	12.60
Patient 16	0.88	0.30	0.22	10.01	21.67	20.15
Patient 17	0.83	0.62	0.78	6.82	8.04	8.19
Patient 18	0.90	0.89	0.87	8.17	8.78	9.87
Patient 19	0.63	0.53	0.49	9.20	14.85	16.98
Patient 20	0.70	0.62	0.88	10.00	15.09	9.54
Patient 21	0.78	0.36	0.52	8.07	22.48	20.21
Patient 22	0.11	0.24	0.87	18.32	17.90	7.35
Patient 23	0.68	0.63	0.82	19.35	21.30	8.25
Patient 24	0.75	0.50	0.58	3.36	12.12	12.16

Mean ± SD	0.73 ± 0.17	0.62 ± 0.19	0.71 ± 0.18	10.19 ± 4.13	13.93 ± 4.38	13.24 ± 3.99

**Table 2:** The area under the receiver operating characteristic curve.

Event	Receiver operating characteristic	
	EACL and SampEn via ANN	BIS and SampEn via ANN
Patient 1	0.996	1.000
Patient 2	0.964	0.999
Patient 3	0.998	0.982
Patient 4	0.980	0.986
Patient 5	0.973	0.897
Patient 6	0.921	0.867
Patient 7	1.000	0.997
Patient 8	0.938	0.999
Patient 9	0.798	0.604
Patient 10	0.987	0.985
Patient 11	0.917	0.964
Patient 12	0.952	0.856
Patient 13	0.999	0.997
Patient 14	0.966	1.000
Patient 15	0.944	0.920
Patient 16	0.988	0.984
Patient 17	0.999	0.993
Patient 18	1.000	0.987
Patient 19	0.964	0.974
Patient 20	0.940	0.946
Patient 21	0.985	0.965
Patient 22	0.807	0.981
Patient 23	0.994	0.896
Patient 24	1.000	0.997

Mean ± SD	0.953 ± 0.07	0.949 ± 0.08
